# New paradigms in pain management after skeletal trauma: Orthopaedic Trauma Association's 2023 Basic Science Focus Forum Symposium

**DOI:** 10.1097/OI9.0000000000000352

**Published:** 2025-04-01

**Authors:** Jasmine V. Hartman Budnik, Thomas F. Higgins, Anne-Marie Malfait, Jarret A.P. Weinrich, Allan I. Basbaum, Joseph R. Hsu, Saam Morshed, Chelsea S. Bahney

**Affiliations:** a Steadman Philippon Research Institute, Vail, CO; b University of Utah, Salt Lake City, UT; c Rush University, Chicago, IL; d Chicago Center on Musculoskeletal Pain, Chicago, IL; e University of California San Francisco, San Francisco, CA; f Wake Forest University, Winston-Salem, NC; g Atrium Health, Charlotte, NC.

**Keywords:** pain management, preclinical models, fracture healing, patient-reported outcomes, orthopaedic trauma

## Abstract

Traumatic injuries are associated with significant acute pain and subsequent high risk of the development of chronic pain. However, addressing pain after skeletal trauma presents a complex challenge to achieve effective pain relief that minimizes risk of addiction and does not interfere with functional recovery. The Orthopaedic Trauma Association's 2023 Basic Science Focus Forum aimed to bridge the gap between basic science and clinical outcomes with an educational symposium on pain management designed to foster collaboration and provide practical strategies from the frontiers of pain research. Owing to the subjective and multifaceted nature of pain, the development of effective preclinical and clinical pain assessment measures is the first step to making impactful progress in studying pain. Preclinical models prove a valuable tool for studying the molecular mechanisms associated with pain following orthopaedic trauma. These models also allow study of the efficacy of novel pain management techniques, such as testing novel analgesics. Translating novel analgesics and pain management strategies to the clinic requires that we have accurate methods to describe pain to determine whether new approaches are meaningful. It is also necessary to recognize the patient's role and the importance of patient education in the prevention of pain medication misuse, particularly in light of the current national opioid crisis. Overall, collaboration with orthopaedic surgeons in the application of these strategies in a clinical setting is vital for addressing the downfalls of current pain management efforts and providing patients with safe and effective improvements in pain relief after skeletal trauma.

## 1. Introduction

Pain is an expected symptom after orthopaedic trauma and plays a protective role in preventing further injury during the initial healing process. However, treating excessive or unresolved pain is of high priority for both patients and physicians when it comes to orthopaedic and postoperative care.^1^ Nevertheless, current pain management options, including nonsteroidal anti-inflammatory drugs (NSAIDs) and opioids, are far from ideal. While the literature is divided on the risks of NSAID use for fracture pain, many studies suggest that they interfere with bone healing.^2–5^ Opioids are another effective method to treat pain, but the high risk of addiction and misuse have contributed to growing calls to address the national opioid crisis. Orthopaedic surgeons are responsible for 7.7% of opioid prescriptions, which is disproportionate to only making up approximately 2.5% of all physicians.^6^ With growing concerns regarding the opioid crisis and delayed fracture healing, there is an urgent need for innovations in developing new solutions for pain treatment, especially in the setting of musculoskeletal trauma. The Orthopaedic Trauma Association's 2023 Basic Science Focus Forum brought together experts in pain research to address this topic as cross-disciplinary discussions are key to improving our understanding of orthopaedic trauma–related pain and furthering advancements in pain management strategies.

## 2. Measuring Pain: From Biometrics to Patient-Reported Outcomes—What Is Useful; What Is Not?

The subjective nature of pain presents a significant challenge to accurate and reproducible pain assessment for both researchers and clinicians, but acute pain and subsequent chronic pain are among the most important drivers of patient outcomes. Pain persistence after trauma is closely related to anxiety and depression and is a key predictor of patient satisfaction after lower extremity surgery.^7,8^ Thirty-five to seventy-five percent of patients with traumatic musculoskeletal injury ultimately develop chronic pain.^9,10^ Effective management of short-term pain after musculoskeletal injury may mitigate the progression to chronic pain, emphasizing the need for effective pain assessment and treatment in the acute phase.^10^ Overall, there is a sequential nature to assessing and addressing pain. To develop the best pain treatment options for patients, it is critical to rigorously study it in a prospective manner. However, to study pain and potentially influence the prevalence of chronic pain, we need to be able to measure it.

Measuring pain starts by defining it and differentiating between subcategories of pain and emotional distress. At the root, somatic pain is the perception of bodily pain, such as from injury of the musculoskeletal system. However, this concept overlaps closely with emotional distress, which encompasses anxiety, depression, fear, and anger. The challenge of isolating measures of somatic pain versus emotional distress is often the complicating factor regarding pain measurement. A key example is the unclear directionality between pain and anxiety after injury. A study on lower extremity trauma showed that while pain weakly predicts anxiety in early stages, there is a stronger relationship from anxiety to pain longitudinally.^8^ These detailed analyses depend heavily on independent measures of somatic pain and emotional distress.

Further complicating the effort to accurately assess pain are variations in the definition and communication of pain in certain subpopulations of patients. Difficulty in obtaining direct subjective reports of pain from children, the elderly, and patients with cognitive or communication impairments limits the accuracy of pain assessment in these populations. While large amounts of psychometric work go into perfecting the wording of pain measurement tools, these efforts do not carry over seamlessly to other languages with simple translation. This is also compounded by differences in norms between cultural and ethnic groups. In a study on pain related to femur fractures, populations from Los Angeles County, United States, and Ho Chi Minh City, Vietnam, showed wide differences in their subjective perception of pain and opiate consumption.^11^ Current efforts to address these differences include the development of country-specific reference values for pain measures such as using the Patient-Reported Outcomes Measurement Information System (PROMIS).^12^

The collection of patient-reported outcomes (PROs) on pain starts with the legacy measures. The Visual Analog Scale (VAS), developed in 1921 by Hayes and Patterson, is a unidimensional, ordinal scale representing the distance between no pain and the worst possible pain. The commonly used Numeric Rating Scale (NRS) is the numerical version of the VAS. Key merits of these scales are that they are easy, fast, repeatable, easy to translate, and can detect small changes. However, the subjective interpretation of pain leads to the scale being more repeatable within an individual rather than between different patients or sets of patients. While it validates well between paper and a laptop, it does not move as seamlessly between a laptop and smartphone.^13^ As an ordinal scale, responses tend to cluster at the extremes so that each unit of the scale does not show an equivalent change, although the more items you add, the more discerning the scale can be.^14^ Most importantly, when it comes to the key question of what the VAS and NRS, long considered the gold standard in pain assessment, actually measure, Clark et al^15^ found that while these scales predict emotional distress, they did not correlate with somatic pain. This is problematic when considering that scales measuring emotional distress may influence decisions to prescribe opiates for somatic pain. Another legacy measure is the Magill Pain Questionnaire, which is often used in clinical trials but increases patient burden because of the large number of questions. Finally, the Brief Pain Inventory (BPI) has gained the most traction in clinical trials as it provides greater detail than an ordinal scale, is concise enough to fit on 2 pages, and can be administered electronically or on paper. Recent studies have shown that the BPI has the strongest psychometric validity, reproducibility, and responsiveness of the legacy measures.^16^

With the goal of decreasing patient burden, there has been a recent shift from legacy measures to computer adaptive testing (CAT). The key measure in this category is the PROMIS Pain Interference (PROMIS PI) bank, which aims to isolate the practical ways that pain changes a patient's life, rather than an overall subjective description of their pain. As a result, the defined aim of the PROMIS PI is to specifically measure pain impact or pain interference, which is “the degree to which pain limits or interferes with an individual's physical, mental, and social activities.”^17^

The difficulty with the subjective nature of pain has also prompted research into trying to identify more objective pain measurement options, such as biomarkers and biometrics. As of now, these are largely unproven but may be avenues for future research into more objective or supplementary pain measurement strategies. Regarding biomarkers, a recent review by Eldabe et al^18^ outlines advances in biomarkers for chronic pain from serum and urine. These include metabolites of inflammation pathways, pain-modulating neurotransmitters, and B6/B12/glutathione depletion. The main downside of using biomarkers to measure pain is the uncertainty of direct correlation. Other options include neuroimaging, biopotentials, and cerebrospinal fluid samples, but these are less repeatable and are better for research rather than being clinically useful at this point. Biometric options, such as wearable electronic devices and EEG measurements, present a similar problem where large amounts of data can be collected but inferred meanings are difficult to link directly to pain.^19,20^ While there remains uncertainty regarding which tools are most useful and effective for pain assessment, continued improvement and validation of pain measurement and management strategies is a necessary foundation for all other areas of pain research.

## 3. Preclinical Models to Measure Pain

Preclinical models in pain research is a useful tool for investigating the underlying biological mechanisms associated with pain and identifying key factors that influence pain development and mitigation. However, to develop quantitative pain assays in preclinical models, rigorous testing and validation is needed to ensure reproducibility and translatability. In the setting of orthopaedic trauma, modeling pain in a preclinical setting also goes hand-in-hand with developing preclinical models and behavioral pain assays specific to musculoskeletal diseases and injury.

Animal models of post-traumatic osteoarthritis (PTOA) are one of the best-used musculoskeletal model systems to research biochemical changes in joint tissues, long-term effects of chronic damage, efficacy of treatment strategies, and stages in the progression of pain. PTOA commonly occurs after intra-articular fracture, chondral damage, or ligamentous injury and leads to problematic changes in neuromuscular control, biomechanics, articular cartilage, and joint loading.^21–23^ A history of joint injury is also linked to 4 times and 5 times greater risk of knee OA and diagnosis of hip OA, respectively.^22^ Pain is the driving clinical symptom associated with PTOA, which is a considerable problem estimated to affect more than 5.6 million people who are seen annually for post-traumatic care.^21^ Using preclinical models to understand how the progression of damage in the joint is related to the progression of pain provides a foundation for making connections between specific biological processes and changes in pain after orthopaedic trauma.

Slowly progressing OA is an important joint pathology to consider because it provides a window into a variety of types and stages of pain, including transient pain, activity-related pain, neuropathic pain (pain resulting from nerve damage), pain that radiates beyond the area of injury, persistent pain, pain flares occurring at rest, and chronic pain. Furthermore, the progressive nature of this pain allows for a detailed analysis of key changes that signal the onset of pain and mark the transition from acute to chronic pain. Preclinical models of slowly progressing OA are a key starting point to understanding how the processes in the joint and the processes of pain are interconnected.

Destabilization of the medial meniscus (DMM) is one common mouse model of PTOA that allows for appropriate assessment of progressive pain-related behaviors.^24,25^ In this model, observed pain-related behaviors include knee hyperalgesia (increased sensitivity to pain), mechanical allodynia (pain response to innocuous stimuli), weight-bearing deficit, changes in locomotion, anxiety-like behaviors, and spontaneous pain observed with conditioned place preference. For knee hyperalgesia, mice showed a decreased threshold for withdrawal from a force on the knee after DMM compared with sham surgery controls, which reflects pain and sensitivity at the knee. With recovery, knee hyperalgesia decreased over a period of 16 weeks after surgery, but DMM mice showed consistently lower withdrawal thresholds than sham mice at each time point.^26^ In a study measuring secondary mechanical allodynia with von Frey fibers, both mice after DMM and sham surgery controls showed a decrease in withdrawal threshold, showing pain sensitization. However, sham mice quickly returned to a baseline threshold while DMM mice maintained hypersensitivity for a full 16 weeks after surgery.^25^ Patterns in weight bearing also change after DMM. For mice that undergo DMM compared with sham surgery controls, both groups offload weight from an operated hindlimb completely for a few days after surgery before returning to a baseline level. However, the behavior of the groups diverges at around 8 weeks, with DMM mice showing a significant decrease in weight bearing on the damaged knee compared with sham mice.^27^ The variety of tests and patterns show that different types of pain behaviors develop in a time-dependent manner, with some happening at early stages versus late stages, with maintenance of these behaviors ranging from transient to sustained. An important, and not yet fully understood, consideration to make within these studies is the influence of age on pain. In a recent study, naïve female and male mice showed significant development of progressive mechanical allodynia, progressive knee hyperalgesia, and a loss of grip strength over a 20-month period as they aged.^28^ As a result, preclinical studies in pain research should use age-matched control animals, especially because longitudinal measurements are key to studying progressive changes in pain-associated behaviors.

There is a wide range of assays to assess pain behaviors in preclinical models with specificity to the type of pain, anatomical target, considerations based on the type of injury, and research goals. Some of the most well-known assays and what they measure include the von Frey test for mechanical allodynia, pressure application measurement for mechanical allodynia, hot/cold plate for thermal sensitivity, rotarod device for motor coordination, horizontal ladder for proprioception, balance beam for proprioception, elevated plus maze for anxiety, conditioned place preference for reinforcement of behaviors, and dynamic weight-bearing tests.^29^ In all these assays, the major challenge is the inability to directly assess pain in preclinical models. Therefore, these assessments need to be designed in a very rigorous manner. Behavioral tests are not one size fits all; each test measures a different aspect of animal behavior and may have unique issues that need to be recognized when implementing them. Careful consideration based on individual study design can ensure the best fit in choosing a pain assay, and it can be useful to incorporate more than one type of measure. While longitudinal testing is preferred, animals should not be tested too often, as some behaviors will change if the animal becomes accustomed/bored with the environment, and one animal should not be subjected to a large number or variety of behavioral assays in one session/experiment because of the stress this puts on the animals. There must be rigorous controls to generate reproducible data with behavioral assessments such as analgesic controls to confirm that a test is pain-related, sham-operated controls, and age-matched naïve groups. The same blinded, experienced tester should be used throughout the experiment. Researchers should also ensure that animals are properly acclimatized to the specific situation and be aware that pharmacologics may have unexpected effects on control animals.

It is important to recognize that the progression of pain is also influenced by genetic differences, mechanical factors such as joint alignment, and systemic factors such as obesity or menopause. Preclinical models to measure pain are an important starting point to understanding the progression of pain after orthopaedic trauma and the factors that influence pain maintenance and recovery. The development of novel methods to measure pain in preclinical models will provide innovative ways to increase sensitivity to changes in pain-associated behaviors. Sadler et al^29^ provide an excellent review of advancements in preclinical pain measurement methods that will be key in defining the future of preclinical pain research. Overall, continued rigorous development and testing of preclinical models for measuring pain will promote high-quality, reproducible, and translatable evidence and advancements in pain research.

## 4. The Quest for Novel Analgesics to Treat Fracture-Related Pain: Where We Start

Pain is commonly associated with fractures, both acutely and in cases of delayed fracture healing or nonunion that clinically presents as nonresolved pain with prolonged evidence of fracture lines in radiographs. Acute, induced pain is an indicator of the fracture and serves an evolutionary role in reducing activity/weight bearing that will exacerbate the injury. During the normal progression of bone healing, the formation of the soft callus helps to stabilize the fracture and presumably reduces the pain to enable a gradual return to weight bearing. This is a critical step in the healing process as loading stimulates bone formation and supports soft-to-hard callus remodeling.

Persistent pain with weight bearing or movement is a key clinical indicator of a poorly healing fracture. Incidence of chronic postfracture pain is significant with reported levels on the low end around 19% for wrist and ankle fractures treated with surgery,^30^ to 55–78% for lower extremity fractures,^31,32^ and as high as 90% following vertebral body fragility fractures.^33^ Patients with impaired healing often experience pain for longer than 6 months, meeting the definition of chronic pain, which stands at “pain that lasts longer than 12 weeks or beyond the natural healing time.”^34^ This becomes increasingly significant when considering the prevalence of fractures, especially in the lower extremity. For 2019, *The Lancet* Global Burden of Diseases reported 178 million new fractures and 445 million prevalent fractures worldwide. Of these, fractures of the patella, tibia/fibula, or ankle were the most burdensome, accounting for 32.7 million cases. Femur fractures accounted for another 14.6 million.^35^ Nonresolving pain associated with delayed healing is the most common reason patients return for medical treatment within 2 years of an initial fracture.^36^

Currently, opioids are the standard-of-care analgesic to treat acute and chronic fracture-related pain across the age span. Practitioners tend to avoid prescribing NSAIDs based on published meta-studies that report they inhibit bone healing.^2^ However, opioid medication misuse is a well-known and alarming problem further complicated by differences in opioid prescription amounts based on type of injury and regional patterns. Patients with pediatric fracture are sent home with an average of 28.4 doses (at recommended mg/kg)^37^ while patients with pelvic fracture are prescribed, on average, 111 oxycodone (5 mg) pills within 90 days postoperatively.^38^ Particularly concerning is that patients taking opioids for longer than 7 days have an increased risk of chronification of pain, and those who are on opioids for upward of 30 days have a high chance of addiction.^39^

To develop and test novel analgesics to address fracture-related pain, it is critical to understand the molecular and cellular mechanisms of pain in both acute induced pain and scenarios where that pain becomes chronic. Furthermore, based on data suggesting that drugs can negatively affect healing, fracture healing outcomes are critical to measure in parallel with pain behaviors. Mechanistic and discovery studies of this nature necessarily begin in preclinical models where the complex interaction of the fracture, immune, and nervous systems can be studied in conjunction with loading behaviors.

Currently, there is a paucity of studies evaluating preclinical postfracture pain, and those that are published use the von Frey up-down method and/or gait analysis.^40–42^ The hindpaw reflexive avoidance assays (Hargreaves, von Frey, Randall–Selitto) have significant limitations when applied to rodent models of fracture healing. These assays are inadequate because the primary site of the pain is localized at the injury, yet the testing site is in the foot where the animal's neuromuscular response is hindered by the presence of a proximal fracture. Alternative methodologies, such as the dynamic weight-bearing assay or gait analyses (CatWalk, DigiGait), are not only insufficiently sensitive to measure induced fracture pain but also labor-intensive, making the techniques unrealistic to use when studying the time course of novel analgesics or potentially short-acting sensitizers. Moreover, these murine protocols often require movements that may not be appropriate for an injured mouse (eg, running on a treadmill or walking across a narrow beam with disrupted gait). These shortcomings emphasize the urgent need for improved and innovative technology to reliably evaluate preclinical pain behavior, from induced to chronic, following fracture.

## 5. New Pain Management Concepts in Fracture

The balance between safety and comfort is a central consideration for addressing pain with current treatment options and exploring new avenues in pain management. With this comes the need to consider how novel pain treatment options affect bone healing and interact with opioid use and misuse. As the problems associated with the opioid epidemic continue to surface, cannabinoids have risen to the forefront of possible alternatives for pain management. However, scientific evidence regarding the medical use of cannabinoids for pain is sparse because of limitations set by federal regulations and restrictions on researching commercial marijuana-based products.^43^ Nevertheless, current research efforts have begun to provide data on the use of cannabinoids for pain, its effect on bone healing, and its relationship to opioid use.

From a pharmacological standpoint, cannabinoids are biological compounds found in the plant *Cannabis sativa*. The 2 most commonly studied compounds of interest when it comes to the reported effects of cannabinoids are tetrahydrocannabinol (THC) and cannabidiol (CBD). THC is the psychoactive component of cannabis and is thought to be responsible for its addictive potential, which may be associated with cannabis use disorder.^44^ CBD is not psychoactive and may be the component responsible for more of the beneficial effects on pain. In general, different strains of cannabis are determined by their ratio of THC and CBD. In recent years, THC content has greatly increased and continues to rise in cannabis samples while CBD content has largely stayed the same.^45^ Beyond THC and CBD, other cannabinoids to consider for further research include cannabichromene and cannabinol.

Overall, a systematic review and meta-analysis of the literature on the medical use of cannabinoids suggests a benefit of cannabinoids in chronic pain treatment with a small-to-moderate effect size.^46^ This is reasonable considering that most studies on pain medication options, including opioids and acetaminophen, also show small-to-moderate effect sizes. While current research suggests a beneficial effect of cannabinoids on pain, there are detailed considerations still to be made. In a review of randomized, double-blind controlled trials, cannabis-based medications showed 50% or greater relief for neuropathic pain compared with a placebo. However, they were associated with more adverse events and a slightly increased incidence of psychiatric disorders, especially in options with high concentrations of THC.^47^ Similarly, cannabis cigarettes containing delta-9-THC have been shown to improve VAS pain scores in a time-dependent manner, although use does come with euphoric effects. In addition, both 3.5% and 7% delta-9-THC cannabis cigarettes were associated with impaired learning and memory, and the 7% delta-9-THC group also experienced impairments in attention and psychomotor speed.^48^

For the most part, current data on the efficacy of cannabinoids for pain come from placebo-controlled studies. However, there is a lack of research directly comparing cannabinoids with other current pain medication options, which illustrates an area for growth in current cannabinoid pain research. Furthermore, it is important to consider interactions between medical cannabis use and opioid misuse. Unfortunately, in a study on chronic opioid users, multivariate analysis showed that cannabis use for pain was significantly associated with increased opioid misuse.^49^ In addition, analysis of prospective data from the National Epidemiological Survey on Alcohol and Related Conditions showed that cannabis use increases the risk of nonmedical opioid use and opioid use disorder 3 years later.^50^ These results show that novel pain medication options cannot be studied in isolation but rather need to be considered in the context of the current pain management environment, including the opioid crisis.

It is also necessary to understand the implications of cannabinoid use for pain on bone healing. The relationship between cannabis use and healthy healing is complex. While evidence suggests that CBD can enhance bone health and metabolism, THC has a counteracting inhibitory effect. Orthopaedic complications associated with marijuana use include delayed bone healing, decreased bone mineral density, new fractures, postoperative hyperemesis, and increased opioid use after orthopaedic surgery.^51^ However, in a study using a preclinical model of tibia fracture, CBD and cannabigerol (CBG) showed decreased pain reactions while also facilitating bone healing mechanisms across the stages of fracture repair, including early promotion of bone progenitors and improved biomechanical quality of healed bone. This suggests that nonpsychotropic cannabinoids such as CBD and CBG may provide novel pain management options that provide effective analgesic effects without adverse effects on fracture healing, as is a problem with NSAIDs.^52^ Overall, the high variability in the route of administration, dosage, cannabinoid content, and method of cannabis use may also contribute to differences in the effects on pain management, health, bone healing, and surgical outcomes. Physicians and researchers need to be aware that nonmedical cannabis use is present in the patient population and may be more prevalent in the orthopaedic trauma population. In a recent retrospective cohort study, the prevalence of cannabis use in patients undergoing surgery or interventional procedures was 14.3% in 2020, with orthopaedics having the highest percentage (25.4%) of patients out of all recreational cannabis use among the studied surgical services.^53^ Therefore, physicians and surgeons need to be able to have open conversations with their patients to make informed decisions about pain management, orthopaedic treatment, and overall health.

Beyond cannabinoids, another novel concept in pain management is aromatherapy, which is the use of essential oils on the skin or through inhalation for therapeutic purposes. Some of the most commonly used plant-derived essential oils for therapeutic use include lavender, eucalyptus, and peppermint.^54^ Aromatherapy has been studied extensively for years, including multiple randomized clinical trials. Results have shown that aromatherapy can have an anxiolytic effect, which should be considered with the demonstrated relationship between anxiety and pain.^55^ Studies also suggest that aromatherapy is better for addressing acute pain rather than chronic pain and has the strongest effect during the postoperative period,^54^ demonstrating that it could serve as a complementary treatment option to address anxiety, pain, and nausea after surgery.^56^

Regardless of what novel pain management strategies are developed through basic science and clinical research, to make a real improvement in the lives of patients, there must be practical, accessible, and applicable resources available for orthopaedic surgeons and health care professionals. Increasing awareness of and effectively distributing patient education tools and evidence-based recommendations for providers is key to furthering progress in pain management efforts and reducing the risk of pain medication misuse. Table 1 lists resources for patients, such as new patient education materials, pain medication agreements, and informational handouts for supplemental pain management options, as well as clinic-facing resources for opioid tapering and pain management strategies. Of note is the Orthopedic Trauma Association's (OTA) Clinical Practice Guidelines (CPG) for Pain Management in Acute Musculoskeletal Injury, which provides practical, scalable, and accessible recommendations in an open-access format for clinicians. By publishing current methodologies, it also identifies gaps in the research to guide future efforts to continue to improve the standard of care for pain management. Education on pain and medication use can be an effective tool to significantly change patients' perceived expectations of pain and influence opiate use.

**Table 1.** Pain Education Resources

Patient pain education resources	
Multimodal pain resources	Link ^58^
Pain agreement, opioid tapers, multimodal painorder set,and patient education materials in English and Spanish	
New patient education materials	Link ^59^
From the orthopaedic surgery research team at Atrium Health Musculoskeletal Institute in line with PRIMUM (prescription reporting with immediate medication utilization mapping) and IMPROVE (implementing a multimodal path to RecOVEry)	
Acute pain management education	Patient Education ^59^
Chronic pain care agreement	Example ^59^
Acute pain management*	Handout ^59^
Compression patient handout*	Handout ^59^
Cryotherapy patient handout*	Handout ^59^
Desensitization patient handout*	Handout ^59^
Guided imagery patient handout*	Handout ^59^
TENS unit guidelines for patients*	Guidelines ^59^
Clinic-facing pain education resources	
Clinical practice guidelines for pain management in acute musculoskeletal injury	Link ^60^
Orthopaedic Trauma Association, forclinicians, includes recommendations for opioid tapering	

* Available in English and Spanish.^59^

## 6. Conclusion

**Figure 1.**
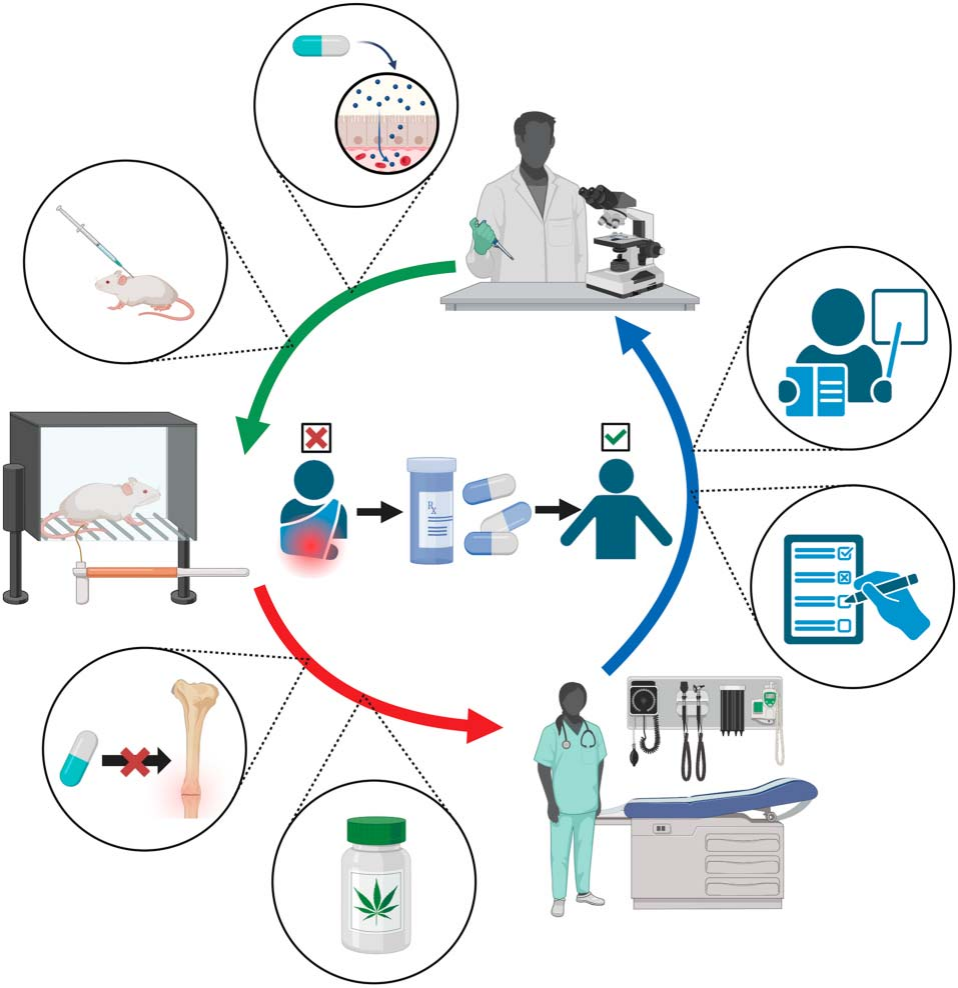
Bench to bedside and back again. Improving pain management strategies involves collaboration between basic science research, studies using preclinical models, and evaluation of clinical applications. At the center of pain management research is the goal to provide patients with safe and effective improvements in pain relief after skeletal trauma. This cycle often starts at the bench, with the investigation of the mechanism of analgesics and drug testing in preclinical models. Next in the cycle is the rigorous testing of preclinical pain assays and the development of preclinical models to help determine the effects of analgesics on fracture healing. This also involves the testing of novel analgesic options, such as cannabinoids, to provide evidence-based suggestions for clinicians regarding new pain management strategies. Research does not stop at the bedside, as patient-reported outcomes and the importance of patient education provide valuable information to build the next waves of innovation in pain research. Altogether, cross-disciplinary collaboration is key to high-quality and impactful findings.

The impact pain has on patient satisfaction and clinical outcomes emphasizes the importance of improving pain measurement and management strategies. Continued improvement of pain assessment methods alongside innovative development and validation of novel options for pain management after musculoskeletal trauma is key to improving orthopaedic patient care. Pain measurement research is the necessary foundation for the subsequent steps of accurately studying pain in both preclinical and clinical settings. Preclinical models specific to orthopaedic trauma can continue to elucidate the biological mechanisms related to pain behaviors, identify targets for novel analgesics, and evaluate the efficacy of pain interventions. While clinicians can put patient education and clinical practice recommendations to use immediately, more research is needed to validate the efficacy and safety of novel pain management options to address the downfalls of current pain management treatments. To promote research in this area, the National Institute of Arthritis and Musculoskeletal and Skin Diseases has recently funded projects that are consistent with the NIH's Helping to End Addiction Long-term initiative to speed scientific solutions to the national opioid public health crisis. Research priorities include understanding the molecular and cellular mechanisms of pain following musculoskeletal injury, identifying patient-specific biomarkers associated with increased susceptibility to chronic pain, and testing treatment options that can effectively alleviate different types of pain. Cross-disciplinary research teams that reach beyond current research bases will be needed to achieve progress in this complex field of musculoskeletal pain.^57^ The collaboration between researchers and orthopaedic surgeons in a “bench to bedside and back again” method, as visualized in Figure 1, is paramount to providing patients with effective, nonaddictive, and evidence-based treatment options to provide patients with better outcomes after musculoskeletal injury.
